# Complete Genome Sequences and Characteristics of Seven Novel Mycobacteriophages Isolated in East Texas

**DOI:** 10.1128/mra.00335-23

**Published:** 2023-06-05

**Authors:** Skylar M. Weiss, Kezia K. Happy, Faith W. Baliraine, Abigail K. Beach, Sean M. Brobston, Claire P. Martinez, Kaitlyn J. Menard, Savannah M. Orton, Angela L. Salazar, Gregory D. Frederick, Frederick N. Baliraine

**Affiliations:** a Department of Biology & Kinesiology, LeTourneau University, Longview, Texas, USA; Portland State University

## Abstract

Full-genome sequences of seven mycobacteriophages isolated from environmental soil samples are presented. These bacteriophages, with their respective clusters or subclusters are Duplo (A2), Dynamo (P1), Gilberta (A11), MaCh (A11), Nikao (K1), Phloss (N), and Skinny (M1). All had siphovirus-like morphologies, with genome sizes ranging from 43,107 to 82,071 bp.

## ANNOUNCEMENT

Bacteriophages are viruses that exclusively infect bacteria, exhibiting obligate intracellular replication and a limited host range (1, 2). Bacteriophages are the most numerous entities in the biosphere, totaling >10^31^ particles (3). The bacterium-bacteriophage relationship exhibits constant bidirectional selective pressure, with bacteria evolving to resist viral infection and bacteriophages coevolving to maintain their replicative ability within their hosts (4, 5). The prevalence of antibiotic-resistant bacterial infections has propelled a resurgent interest in phage therapy (6–10). Here, we report seven novel lysogenic bacteriophages.

**FIG 1:**
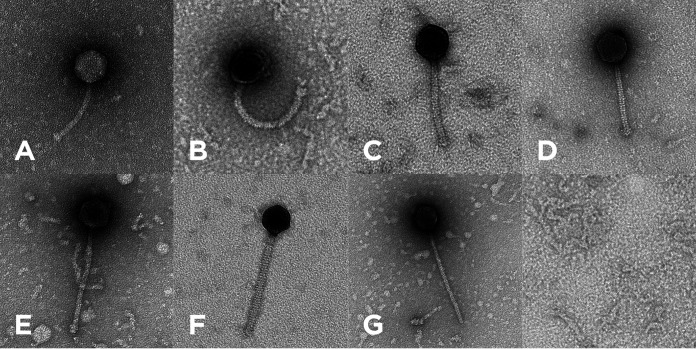
Transmission electron micrographs of the seven bacteriophages, Duplo (A), Dynamo (B), Gilberta (C), MaCh (D), Nikao (E), Phloss (F), and Skinny (G). Capsid sizes and tail lengths are provided in Table 1. Bacteriophage particles were added to 300-mesh carbon–Formvar-coated copper grids (Ted Pella Inc., Redding, CA), stained with 1% (wt/vol) uranyl acetate, and imaged at the University of Arkansas for Medical Sciences Digital Microscopy Laboratory.

All bacteriophages were isolated from environmental soil samples collected from various locations in east Texas during 2020 to 2021 (Table 1), using standard methods (5). In short, the soil samples were washed in Middlebrook 7H9 medium prior to centrifugation and supernatant filtration (pore size, 0.22 μm). The filtrates were subsequently inoculated with Mycobacterium smegmatis mc^2^155 cells and incubated at 25°C for 3.5 days with shaking. Filtered samples of each culture were plated with M. smegmatis cells in 7H9 top agar. After purification through three rounds of plating at 37°C for 48 h, the observed plaque morphologies of the various phages ranged from clear to turbid (Table 1). Negative-stain transmission electron microscopy showed all these bacteriophages to exhibit a siphovirus morphotype, with isometric capsids (diameter, ~51.76 to 71.53 nm) and noncontractile tails (length, ~124.23 to 326.19 nm) (Fig. 1 and Table 1), measured using ImageJ (11–13).

**TABLE 1:** Properties of seven mycobacteriophages isolated in east Texas, USA, in 2020 and 2021

Characteristic	Data for phage:^*a*^						
	Duplo	Dynamo	Gilberta	MaCh	Nikao	Phloss	Skinny
Yr found	2020	2021	2021	2020	2020	2020	2021
Location found	Big Sandy, TX	Longview, TX	Longview, TX	Longview, TX	Longview, TX	Longview, TX	Longview, TX
Soil sampling location	32.588034 N,95.063959 W	32.46402 N,94.728226 W	32.468338 N,94.726844 W	32.465237 N,94.727035 W	32.54954 N, 94.821309 W	32.4675 N, 94.725 W	32.467601 N,94.723749 W
Lysate titer (PFU/mL)	3.0 × 10^9^	1.0 × 10^10^	1.0 × 10^9^	1.6 × 10^10^	2.17 × 10^9^	8.7 × 10^11^	1.18 × 10^9^
Plaque morphology after 48 h at 37°C	Slightly turbid with defined edges	Clear with defined edges	Clear with defined edges	Clear with defined edges	Slightly turbid with defined edges	Clear with defined edges	Clear with defined edges
Plaque size (mm)	3.3	0.93	1.5	2.7	1.25	2.0	1.0
Approx coverage (×)	1,516	1,499	5,472	705	414	2,005	499
Genome size (bp)	52,781	46,673	51,470	52,616	59,052	43,107	82,071
GC content (%)	63.5	67.2	63.7	63.6	67.2	66.3	61.5
Overhang sequence	CGGTCGGTTA	CCCGCCCCCCGA	CGGTCGGTTA	CGGTCGGTTA	CTCGGGGGCAT	CCCGCCGCAATGG	ACCTCCTGCAA
Overhang length (bases)	10	12	10	10	11	13	11
Cluster	A	P	A	A	K	N	M
Subcluster	A2	P1	A11	A11	K1		M1
GenBank accession no.	OP297553	OP434454	OP297532	OP297549	OP297530	OP297540	OP297551
SRA accession no.	SRX19690833	SRX19690834	SRX19690836	SRX19690844	SRX19690847	SRX19690850	SRX19690858
Total no. of reads	560,502	485,417	1,968,556	261,625	172,343	608,118	285,773
No. of predicted genes	99	78	99	100	80	70	163
No. of predicted tRNAs	5	0	1	1	0	0	18
tRNA type(s)	Asn, Gln, Glu, Trp, Tyr		Trp	Trp			Trp, Asn, other (Arg/Ala), Tyr, Gln, Pro, Ser, Phe, Met, Arg, His, Leu, Lys, Gly, Val, Thr, Asp, Glu
No. of genes with predicted functions	33	32	36	43	41	28	46
% of genes with predicted functions	33	41	36	43	51	40	28
Key predicted lysogenic life cycle genes	Integrase, excise, immunity repressor	Integrase, excise, immunity repressor	Integrase, immunity repressor	Immunity repressor	Integrase, excise, immunity repressor	Integrase, excise, immunity repressor	Integrase
No. of orphams	2	0	1	0	0	0	2
Capsid size (nm [*n* value])	64.48 (9)	60.90 (7)	52.09 (3)	58.67 (21)	67.04 (5)	51.76 (7)	71.53 (3)
Avg tail length (nm [*n* value])	124.23 (9)	205.57 (6)	125.54 (3)	130.93 (21)	210.16 (5)	172.04 (7)	326.19 (3)
Isolated by	Skylar M. Weiss, Jimena H. Segovia	Christina A. Holder, Kaitlyn J. Menard, Brady E. Tyler	Hattie R. Mills, Ashlyn B. Collier, Kalista J. Rivera, Claire P. Martinez	Matthew S. Adams, Camryn L. Hill	Kezia K. Happy	Summer L. Apostalo, Gavin J. Meyer	Jenna F. Curran, Kristen N. Rose

a All phages were isolated using the enriched isolation method (5) and purified through three sequential 48-h rounds of plating at 37°C. The genomes were sequenced using the Illumina shotgun sequencing method with 150-base single-end reads using the NEB Ultra II Library sequencing kit. All had 3′ sticky overhang genome ends. Genomic termini were identified through buildups of read start positions and variations in genome-wide coverage and verified manually using Consed v29 (14, 15). All phages had a siphovirus morphotype and were predicted to be temperate based on the presence of predicted lysogeny-related genes.

Genomic DNA was extracted from lysates of various titers (Table 1) using the Promega Wizard DNA cleanup kit. Preparation for sequencing using the Illumina MiSeq platform (v3 reagents) was conducted with the NEBNext Ultra II library prep kit. Assembly and verification of the untrimmed reads were performed using Newbler v2.9 (16) and Consed v29 (14, 15). Sequencing revealed genomes ranging in length from 43,107 bp (phage Phloss) to 82,071 bp (phage Skinny) (Table 1). All had 3′ sticky overhangs (10 to 13 bp long) and an average GC content of 64.7% (range, 61.5% to 67.2%), comparable to the 67.4% GC content of their isolation host, Mycobacterium smegmatis mc^2^155 (17). The seven phages were assigned to subclusters A2, A11, K1, M1, and P1 and cluster N (Table 1) based on ≥35% gene content similarity (GCS) to other phages, using the GCS tool in PhagesDB (18, 19).

Genome annotation was accomplished using DNA Master v5.23.6; Starterator; Phamerator (20); BLASTp with NCBI GenBank and PhagesDB (21, 22); GeneMark v2.5p (23); HHpred, with the PDB_mmCIF70_17_Apr, Pfam-A_v35, UniProt-SwissProt-viral70_3_Nov_2021, and NCBI_Conserved_Domains_v3.19 databases (24, 25); Glimmer v3.02 (26); TMHMM v.2.0 (27); SOSUI v1.11 (28); tRNAscan-SE v2.0 (29, 30); and ARAGORN v1.2.41 (31). Default settings were used for all programs (32). An average of 98.0 putative protein-coding genes (range, 70 to 163) and 3.6 tRNAs (range, 0 to 18) were predicted (Table 1). Functions could only be predicted for 28% to 51% of the putative genes in the phages (Table 1). All phages had at least one of the three key genes associated with a lysogenic life cycle. Duplo, Dynamo, Nikao, and Phloss had the integrase, excise, and immunity repressor genes; Gilberta had both the integrase and immunity repressor genes, while Skinny and MaCh had only the integrase and immunity repressor genes, respectively (Table 1).

### Data availability.

The raw reads of all seven reported mycobacteriophages are available in the Sequence Read Archive (SRA), and their complete genome sequences are available at GenBank. The SRA and GenBank accession numbers are provided in Table 1. High-titer lysates of the phages are archived at the University of Pittsburgh Bacteriophage Institute.

## References

[1] Jacobs-Sera D, Marinelli LJ, Bowman C, Broussard GW, Guerrero Bustamante C, Boyle MM, Petrova ZO, Dedrick RM, Pope WH, Modlin RL, Hendrix RW, Hatfull GF, Science Education Alliance Phage Hunters Advancing Genomics and Evolutionary Science (SEA-PHAGES) Program. 2012. On the nature of mycobacteriophage diversity and host preference. Virology 434:187–201. doi:10.1016/j.virol.2012.09.026. 23084079 PMC3518647

[2] Boeckaerts D, Stock M, Criel B, Gerstmans H, De Baets B, Briers Y. 2021. Predicting bacteriophage hosts based on sequences of annotated receptor-binding proteins. Sci Rep 11:1467. doi:10.1038/s41598-021-81063-4. 33446856 PMC7809048

[3] Comeau AM, Hatfull GF, Krisch HM, Lindell D, Mann NH, Prangishvili D. 2008. Exploring the prokaryotic virosphere. Res Microbiol 159:306–313. doi:10.1016/j.resmic.2008.05.001. 18639443

[4] Naureen Z, Dautaj A, Anpilogov K, Camilleri G, Dhuli K, Tanzi B, Maltese PE, Cristofoli F, De Antoni L, Beccari T, Dundar M, Bertelli M. 2020. Bacteriophages presence in nature and their role in the natural selection of bacterial populations. Acta Biomed 91:e2020024. doi:10.23750/abm.v91i13-S.10819. PMC8023132 33170167

[5] Poxleitner M, Pope W, Jacobs-Sera D, Sivanathan V, Graham H. 2018. Phage discovery guide. Howard Hughes Medical Institute, Chevy Chase, MD. https://seaphagesphagediscoveryguide.helpdocsonline.com/home. Accessed 12 May 2023.

[6] Azam AH, Tan XE, Veeranarayanan S, Kiga K, Cui L. 2021. Bacteriophage technology and modern medicine. Antibiotics 10:999. doi:10.3390/antibiotics10080999. 34439049 PMC8388951

[7] Chen Y, Batra H, Dong J, Chen C, Rao VB, Tao P. 2019. Genetic engineering of bacteriophages against infectious diseases. Front Microbiol 10:954. doi:10.3389/fmicb.2019.00954. 31130936 PMC6509161

[8] Hatfull GF, Dedrick RM, Schooley RT. 2022. Phage therapy for antibiotic-resistant bacterial infections. Annu Rev Med 73:197–211. doi:10.1146/annurev-med-080219-122208. 34428079

[9] Dedrick RM, Guerrero-Bustamante CA, Garlena RA, Russell DA, Ford K, Harris K, Gilmour KC, Soothill J, Jacobs-Sera D, Schooley RT, Hatfull GF, Spencer H. 2019. Engineered bacteriophages for treatment of a patient with a disseminated drug-resistant Mycobacterium abscessus. Nat Med 25:730–733. doi:10.1038/s41591-019-0437-z. 31068712 PMC6557439

[10] Dedrick RM, Smith BE, Cristinziano M, Freeman KG, Jacobs-Sera D, Belessis Y, Whitney Brown A, Cohen KA, Davidson RM, van Duin D, Gainey A, Garcia CB, Robert George CR, Haidar G, Ip W, Iredell J, Khatami A, Little JS, Malmivaara K, McMullan BJ, Michalik DE, Moscatelli A, Nick JA, Tupayachi Ortiz MG, Polenakovik HM, Robinson PD, Skurnik M, Solomon DA, Soothill J, Spencer H, Wark P, Worth A, Schooley RT, Benson CA, Hatfull GF. 2023. Phage therapy of Mycobacterium infections: compassionate use of phages in 20 patients with drug-resistant mycobacterial disease. Clin Infect Dis 76:103–112. doi:10.1093/cid/ciac453. 35676823 PMC9825826

[11] Schneider CA, Rasband WS, Eliceiri KW. 2012. NIH Image to ImageJ: 25 years of image analysis. Nat Methods 9:671–675. doi:10.1038/nmeth.2089. 22930834 PMC5554542

[12] Schindelin J, Arganda-Carreras I, Frise E, Kaynig V, Longair M, Pietzsch T, Preibisch S, Rueden C, Saalfeld S, Schmid B, Tinevez JY, White DJ, Hartenstein V, Eliceiri K, Tomancak P, Cardona A. 2012. Fiji: an open-source platform for biological-image analysis. Nat Methods 9:676–682. doi:10.1038/nmeth.2019. 22743772 PMC3855844

[13] Rueden CT, Schindelin J, Hiner MC, DeZonia BE, Walter AE, Arena ET, Eliceiri KW. 2017. ImageJ2: ImageJ for the next generation of scientific image data. BMC Bioinformatics 18:529. doi:10.1186/s12859-017-1934-z. 29187165 PMC5708080

[14] Gordon D, Green P. 2013. Consed: a graphical editor for next-generation sequencing. Bioinformatics 29:2936–2937. doi:10.1093/bioinformatics/btt515. 23995391 PMC3810858

[15] Russell DA. 2018. Sequencing, assembling, and finishing complete bacteriophage genomes. Methods Mol Biol 1681:109–125. doi:10.1007/978-1-4939-7343-9_9. 29134591

[16] Margulies M, Egholm M, Altman WE, Attiya S, Bader JS, Bemben LA, Berka J, Braverman MS, Chen Y-J, Chen Z, Dewell SB, Du L, Fierro JM, Gomes XV, Godwin BC, He W, Helgesen S, Ho CH, Irzyk GP, Jando SC, Alenquer MLI, Jarvie TP, Jirage KB, Kim J-B, Knight JR, Lanza JR, Leamon JH, Lefkowitz SM, Lei M, Li J, Lohman KL, Lu H, Makhijani VB, McDade KE, McKenna MP, Myers EW, Nickerson E, Nobile JR, Plant R, Puc BP, Ronan MT, Roth GT, Sarkis GJ, Simons JF, Simpson JW, Srinivasan M, Tartaro KR, Tomasz A, Vogt KA, Volkmer GA, et al. 2005. Genome sequencing in microfabricated high-density picolitre reactors. Nature 437:376–380. doi:10.1038/nature03959. 16056220 PMC1464427

[17] Mohan A, Padiadpu J, Baloni P, Chandra N. 2015. Complete genome sequences of a Mycobacterium smegmatis laboratory strain (MC2 155) and isoniazid-resistant (4XR1/R2) mutant strains. Genome Announc 3:e01520-14. doi:10.1128/genomeA.01520-14. 25657281 PMC4319614

[18] Russell DA, Hatfull GF. 2017. PhagesDB: the actinobacteriophage database. Bioinformatics 33:784–786. doi:10.1093/bioinformatics/btw711. 28365761 PMC5860397

[19] Pope WH, Mavrich TN, Garlena RA, Guerrero-Bustamante CA, Jacobs-Sera D, Montgomery MT, Russell DA, Warner MH, Hatfull GF, Science Education Alliance-Phage Hunters Advancing Genomics and Evolutionary Science (SEA-PHAGES). 2017. Bacteriophages of Gordonia spp. display a spectrum of diversity and genetic relationships. mBio 8:e01069-17. doi:10.1128/mBio.01069-17. 28811342 PMC5559632

[20] Cresawn SG, Bogel M, Day N, Jacobs-Sera D, Hendrix RW, Hatfull GF. 2011. Phamerator: a bioinformatic tool for comparative bacteriophage genomics. BMC Bioinformatics 12:395. doi:10.1186/1471-2105-12-395. 21991981 PMC3233612

[21] Altschul SF, Gish W, Miller W, Myers EW, Lipman DJ. 1990. Basic local alignment search tool. J Mol Biol 215:403–410. doi:10.1016/S0022-2836(05)80360-2. 2231712

[22] Ismail HD. 2022. Bioinformatics: a practical guide to NCBI databases and sequence alignments. Chapman and Hall, New York, NY.

[23] Borodovsky M, McIninch J. 1993. Recognition of genes in DNA sequence with ambiguities. Biosystems 30:161–171. doi:10.1016/0303-2647(93)90068-n. 8374073

[24] Zimmermann L, Stephens A, Nam SZ, Rau D, Kübler J, Lozajic M, Gabler F, Söding J, Lupas AN, Alva V. 2018. A completely reimplemented MPI Bioinformatics Toolkit with a new HHpred server at its core. J Mol Biol 430:2237–2243. doi:10.1016/j.jmb.2017.12.007. 29258817

[25] Gabler F, Nam SZ, Till S, Mirdita M, Steinegger M, Söding J, Lupas AN, Alva V. 2020. Protein sequence analysis using the MPI Bioinformatics Toolkit. Curr Protoc Bioinformatics 72:e108. doi:10.1002/cpbi.108. 33315308

[26] Delcher AL, Harmon D, Kasif S, White O, Salzberg SL. 1999. Improved microbial gene identification with GLIMMER. Nucleic Acids Res 27:4636–4641. doi:10.1093/nar/27.23.4636. 10556321 PMC148753

[27] Krogh A, Larsson B, Von Heijne G, Sonnhammer ELL. 2001. Predicting transmembrane protein topology with a hidden Markov model: application to complete genomes. J Mol Biol 305:567–580. doi:10.1006/jmbi.2000.4315. 11152613

[28] Hirokawa T, Boon-Chieng S, Mitaku S. 1998. SOSUI: classification and secondary structure prediction system for membrane proteins. Bioinformatics 14:378–379. doi:10.1093/bioinformatics/14.4.378. 9632836

[29] Lowe TM, Chan PP. 2016. tRNAscan-SE On-line: integrating search and context for analysis of transfer RNA genes. Nucleic Acids Res 44:W54–W57. doi:10.1093/nar/gkw413. 27174935 PMC4987944

[30] Chan PP, Lin BY, Mak AJ, Lowe TM. 2021. TRNAscan-SE 2.0: improved detection and functional classification of transfer RNA genes. Nucleic Acids Res 49:9077–9096. doi:10.1093/nar/gkab688. 34417604 PMC8450103

[31] Laslett D, Canback B. 2004. ARAGORN, a program to detect tRNA genes and tmRNA genes in nucleotide sequences. Nucleic Acids Res 32:11–16. doi:10.1093/nar/gkh152. 14704338 PMC373265

[32] Pope W, Jacobs-Sera D, Russell D, Cresawn S, Hatfull G. 2017. SEA-PHAGES bioinformatics guide. https://seaphagesbioinformatics.helpdocsonline.com/home. Accessed 12 May 2023.

